# Intrapersonal Behavioral Coordination and Expressive Accuracy During First Impressions

**DOI:** 10.1177/19485506211011317

**Published:** 2021-04-28

**Authors:** Nida Latif, Lauren J. Human, Francesca Capozzi, Jelena Ristic

**Affiliations:** 1 School of Communication Sciences and Disorders, 5620 McGill University, Montreal, Quebec, Canada; 2 Department of Psychology, 5620 McGill University, Montreal, Quebec, Canada; * Joint first authors

**Keywords:** first impressions, expressive accuracy, intrapersonal behavioral coordination variability, nonverbal behavior, personality judgments

## Abstract

What factors influence how accurately we express our personalities? Here, we investigated the role of targets’ nonverbal expressivity or the intrapersonal coordination between head and body movements. To do so, using a novel movement quantification method, we examined whether variability in a person’s behavioral coordination was related to how accurately their personality was perceived by naive observers. Targets who exhibited greater variability in intrapersonal behavior coordination, indicating more expressive behavior, were perceived more accurately on high observability personality items, such as how energetic and helpful they are. Moreover, these associations held controlling for other indicators of overall movement, self- and perceiver-rated extroversion, as well as how engaging and likable targets were perceived to be. This provides preliminary evidence that variability in intrapersonal behavioral coordination may be a unique behavioral indicator of expressive accuracy, although further research that replicates these findings and examines the causal associations is needed.

Perceiving others’ personalities accurately is important for interactions and relationships (Funder, 1995; Human et al., 2020; Weidman et al., 2015). This is a challenging feat, as there is great variability in how accurately people express their personalities, an important factor in personality impression accuracy (Human & Biesanz, 2013; Rogers & Biesanz, 2019). That is, there are large individual differences in *expressive accuracy* (Human & Biesanz, 2013), the tendency to be accurately perceived, also termed *judgeability* (Colvin, 1993), or being a *good target* (Funder, 1995). Although multiple factors can influence expressive accuracy, past research has primarily examined the role of broad individual differences, such as psychological well-being (e.g., Human et al., 2014; Human et al., 2019) and personality traits like extroversion (Colvin, 1993; Human et al., 2021). Here, we examine the role of *intrapersonal behavioral coordination* (IBC), or the degree of movement coordination between the head and body within a person, as an indicator of nonverbal expressivity. In doing so, we present one of the first examinations of the relationship between a concrete behavioral tendency and personality perception accuracy.

## What Is IBC?

**Figure 1.**
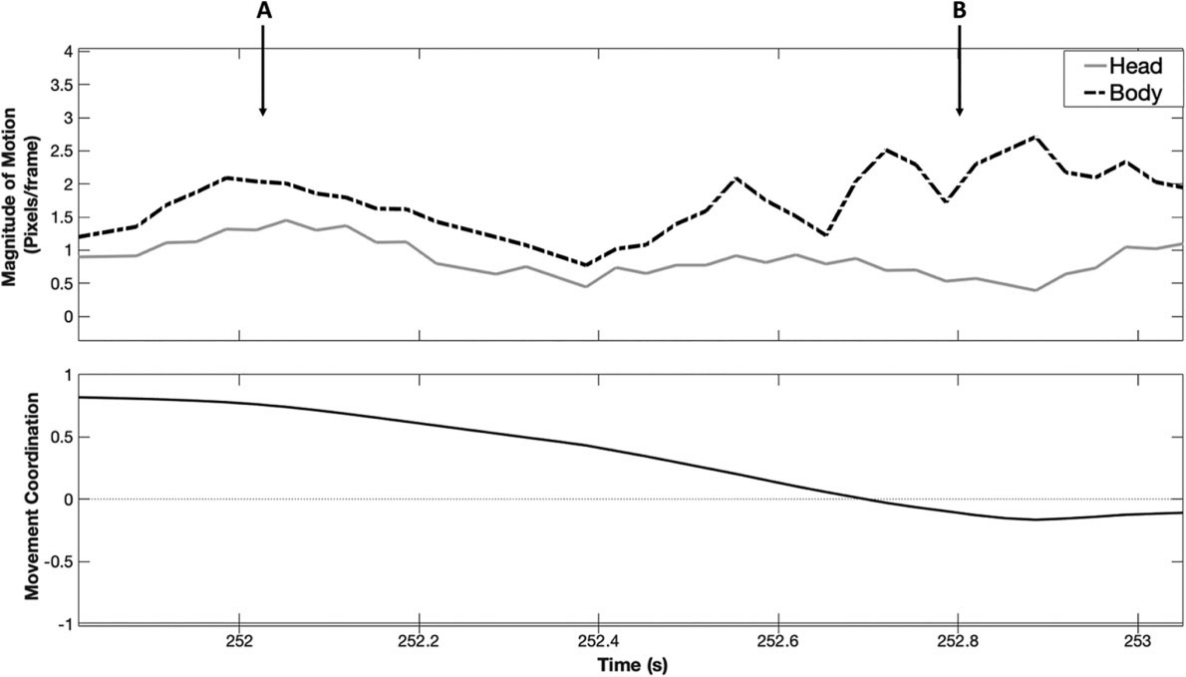
Movement of the head and body over time of an individual as they communicate with another individual. *Note*. The top panel shows variation in the amount of overall movement of the head (gray line) and body (black dashed line) in pixels/frame (see Method section for details on the unit). The bottom panel shows the coordination between these two regions, where 0 indicates no coordination, +1 indicates perfect coordination. While an individual communicates, the coordination between these two regions varies dynamically. When there is greater similarity in movement of the head and body regions, there is greater intrapersonal behavioral coordination (A), and when there is less similarity between these regions, there is lower intrapersonal behavioral coordination (B).

IBC is a measure of the consistency in motion between a person’s head and body. To illustrate, Figure 1 (top) plots the magnitude of motion of a person’s head (solid) and body (dashed line) while talking to another individual. Here, for example, a person may nod their head while speaking, while also either gesturing with their hands (i.e., high coordination) or keeping their hands still (e.g., lower coordination). The bottom panel illustrates dynamic variability in IBC with 0 point denoting no coordination and +1 denoting perfect coordination. Thus, from the magnitude of motion, one can extract a person’s overall *level* of IBC or how coordinated their head and body movements were across the interaction, and *variability* in IBC or how much their level of coordinated head and body movement changed during the interaction.

## What Does It Mean to Be Accurately Perceived?

We take a realistic accuracy approach (Funder, 1995), which defines expressive accuracy as the extent to which a person (i.e., target) is perceived in line with realistic indicators like self- and close-other reports of their personality. Specifically, we take a profile approach, examining the extent to which a target’s ordering on personality items is understood—for example, whether a target is recognized to be more kind than organized and more organized than anxious. Equivalently, this approach indicates whether a person’s level on each item is higher or lower than others (e.g., if a person is kinder than others), on average across items (Biesanz, 2020). Importantly, we also distinguish between distinctive and normative accuracy (Biesanz, 2010; Cronbach, 1955; Furr, 2008). Normative accuracy refers to accurately perceiving how a person is similar to the average person. Distinctive accuracy refers to accurately perceiving how a person is different from the average person. Although normative accuracy can contribute to accurate impressions, it is unclear whether it is determined by knowledge of the target or by a more general normative knowledge. Furthermore, as the normative profile is typically highly socially desirable (Wood & Furr, 2015), it can also imply highly positive impressions. As such, here, we focus on distinctive accuracy as this aspect of expressive accuracy is more likely driven by the understanding of the target. We use the terms distinctive accuracy and accuracy interchangeably.

According to the realistic accuracy model (RAM), to be accurately perceived, targets need to make *relevant* cues *available* to perceivers, who then need to *detect* and *utilize* those cues. Targets’ characteristics that facilitate any of these stages promote expressive accuracy. For example, psychological adjustment may foster expressive accuracy by enhancing cue relevance, as it has been associated with higher personality–behavior congruence or behaving in line with one’s distinctive personality (Human et al., 2014; Human et al., 2019). Similarly, extroversion may also facilitate expressive accuracy by enhancing cue relevance, availability, and/or detection (Colvin, 1993; Human et al., 2021). However, past research has focused on macro psychological processes that do not provide concrete information regarding what good targets do behaviorally to facilitate accuracy. Here, we examine IBC as a microlevel behavioral correlate of expressive accuracy.

## Why Is IBC Informative for Understanding Expressive Accuracy?

Head and body movements play an important role in social communication. Movements within the head region, including facial expressions, mouth, and head movements (e.g., nodding), support our ability to communicate with others, both for verbal speech content (e.g., Munhall et al., 2004) and nonverbal signaling of emotions and attitudes (Wagner et al., 2014). Body movements (e.g., arm movements, postural changes), or “gestures” that accompany speech, also play an important role in understanding social content, demonstrating attitudes, attentional engagement, and agreement (Wagner et al., 2014). Head movements produced while we speak, including mouth movements and gross movements such as head tilts, are coupled in time with gestural body movements (Barbosa et al., 2007; Danner et al., 2018). The temporal coordination between head and body movements, or IBC, and the variability of IBC within a person both play important roles in efficient communication (Wagner et al., 2014). For example, greater IBC is observed when the meaning of what is said is emphasized whereas greater variability in IBC may emerge when socially relevant information, such as attitudes or familiarity, is being additionally conveyed (Wagner et al., 2014).

Overall, then, IBC may represent an important nonverbal behavioral indicator of the degree of personality expressivity. Indeed, the movements involved have been associated with perceptions and self-reports of traits that indicate greater expressivity. For example, overall quantity and variability of head and body movements have been associated with attributing stick figures with traits such as dominance and aggressiveness (Koppensteiner et al., 2017) while people who gesture more self-report greater extroversion (Hostetter & Potthoff, 2012).

In addition to the overall amount of movement of one’s head and body, indicators of the coordination between these movements have been associated with perceptions of particular personality traits that relate to nonverbal expressivity. While IBC is observed due to the strong coupling between speech and gesture in general, its manifestations have been found to influence perceptions of personality traits. For example, virtual agents displaying greater IBC and reduced independent movement of the head and body led observers to attribute those movement patterns to an introverted personality trait (Celiktutan & Gunes, 2015). In contrast, agents showing greater variability in IBC, or faster and more dynamic changes in coordination, where the head and body moved independently, were associated with more extroverted personality traits (Neff et al., 2010).

Importantly, the majority of this past work has examined the links between body movements and perceived personality traits using virtual agents or artificial objects. Whether naturalistic IBC is related to actual or perceived personality or to the accuracy of personality, expression remains poorly understood. Additionally, people with a high level of IBC are likely to be less variable because a person’s head and body movements tend to be highly related, leaving little room for variability in their coupling. Thus, it also remains unclear whether IBC level and variability independently relate to social skill and expressivity, and potential correlates such as extroversion and expressive accuracy. In the present study, IBC levels and variability were highly correlated and only IBC variability had independent associations with expressive accuracy when both variability and level were examined together (see Supplemental Online Materials [SOM]). As such, we focus on IBC variability for the remainder of the manuscript.

## How Does Measuring IBC Inform Current Models of Personality Expression?

Greater IBC variability might suggest a more expressive, socially skillful behavioral style that could result in the greater relevance, availability, and detection of personality cues in turn promoting expressive accuracy. This is consistent with evidence that extroversion is associated with being a good target (Ambady et al., 1995; Colvin, 1993; Human et al., 2021), as it too may imply a behavioral style that facilitates expressive accuracy through each of these pathways. However, extroversion is a broad trait with multiple facets, not all of which might be directly relevant to expressive accuracy. As such, IBC variability may be a more precise predictor of expressive accuracy, shedding more direct light on a specific behavioral profile that could contribute to being a good target.

IBC variability however may not simply signal that a person is extroverted, although it certainly may be a valid cue. Instead, it may more generally enable a perceiver to understand a target’s profile across numerous personality traits because it might make more relevant behavioral cues available to perceivers, some of which may reveal levels of extroversion (both high and low) and some of which that may reveal other traits (such as agreeableness or intelligence). Similarly, if IBC variability elicits greater perceiver cue detection through a more socially skillful and engaging interpersonal style, this should facilitate accuracy for a range of personality traits, not just indicate greater extroversion. In particular, the association with IBC variability may be relevant to the expressive accuracy of personality traits that have clear behavioral manifestations, specifically, those that are high in observability (Funder & Dobroth, 1987; Krzyzaniak & Letzring, 2019). High observability traits, such as extroversion and intelligence, are readily available to others with clear external cues, whereas lower observability traits, such as neuroticism, can remain within one’s subjective experience and may not always manifest externally (John & Robins, 1993; Vazire, 2010). As such, as a nonverbal behavioral characteristic, IBC variability is less likely to relate to the accuracy of impressions for less observable traits, such as neuroticism, which may be better revealed via verbal behavior (e.g., sharing one’s thoughts and feelings). Thus, IBC variability may relate to expressive accuracy more strongly for high observability traits than low observability traits. This is in line with recent findings that extroversion is more strongly related to expressive accuracy for high observability traits (Human et al., 2021).

In sum, in this study, we examined how variability in IBC between individuals’ head and body related to their expressive accuracy during first impressions. In particular, we examined whether IBC variability was associated with how accurately one’s personality was perceived by naive observers for both high and low observability personality items. In addition, in supplemental analyses, we examine how IBC variability relates to other indicators of motion, including head and body motion and variability, and traits and characteristics related to expressivity and social skill, including self- and perceiver-rated extroversion and perceiver-rated engagement and liking. This allowed us to both shed greater conceptual light on the meaning of naturalistically observed IBC variability and to determine the robustness of any associations with expressive accuracy above and beyond these correlates (see SOM).

## Method

We report all data exclusions, conditions, all variables related to the present research questions, and sample size determinations below. A separate analysis on a subset of the participants used here has been previously published (Capozzi et al., 2020).^1^ The analyses in the present study were exploratory and not preregistered and we did not correct for multiple testing; as such, the present results are preliminary. All procedures were approved by the university’s ethics board. Participants were compensated with extra course credit or cash.

### Participants

#### Targets

Participants at least 18 years of age were recruited from a university to serve as targets as part of a larger study on forming personality impressions.^2^ Our goal was to recruit a minimum of 100 targets with complete data or as many as possible between September 2015 and April 2016.^3^ The final data set included 105 targets (*M*_age_ = 20.75, *SD*_age_ = 2.23, 84% female). The study was not designed to address the present research questions but expected power analyses indicate that statistical power was sufficient to do so. To calculate expected power, we used an effect size estimate from past research using a similar video perception paradigm to examine the association between expressive accuracy and target psychological adjustment (Human et al., 2014), which had a moderate association (estimated *r* = .32). After incorporating the uncertainty of the initial effect size estimate into the analysis, the expected power with a sample of 105 targets was .86 (see Biesanz & Schrager, 2017; McShane & Bockenholt, 2015; fabs package for R: github\jbiesanz\fabs).^4^

#### Perceivers

A separate set of participants at least 18 years of age were recruited from the university to serve as perceivers. Our goal was to recruit at least 80 perceivers or as many as possible between June 2016 and December 2016 in order to have at least 10 perceivers view each target. A total of 94 perceivers participated (*M*age = 23.98, *SD*age = 7.98, 30 males, 62 females, and two others), with 10–16 perceivers viewing each target.

### Procedure and Measures

#### Expressive accuracy

Upon arrival, targets provided consent and completed an initial questionnaire that included personality self-reports on the 44-item Big Five Inventory (BFI; Benet-Martinez & John, 1998) plus three items assessing intelligence, for example, “Is bright” (Human & Biesanz, 2011), on a 1 (*strongly disagree*) to 7 (*strongly agree*) scale. Targets also provided the contact information of up to three family members or friends (close others) to complete the BFI about the target to average together with the targets’ self-report to serve as the accuracy criterion. The questionnaire was emailed to close others and responses were received from 135 individuals (96 females, *M*_age_ = 28.11, *SD*_age_ = 13.15), resulting in 77 targets with at least one close-other report. When more than one close-other report was received, the reports were first averaged together and then averaged with the target’s self-report to serve as the accuracy criterion. For targets without a close-other report, only their self-report was used for their accuracy criterion. A subset of 21 items from the BFI (Benet-Martinez & John, 1998) plus the three intelligence items was used for the accuracy criterion to correspond to the subset of items that perceivers rated targets on (see below). The numbers of the specific items used from the BFI-44 are listed in Human and Biesanz (2011).

Following the initial questionnaire, targets completed a video-recorded interview with a female research assistant (interviewer). Targets were asked 10 questions by the interviewer and the full interview took 3–10 min depending on the length of targets’ responses. To ease the perceiver burden, target videos were edited to include responses to two questions that have been used in prior research (Human et al., 2012). These questions were the following: “What are two or three things you do in your leisure time?” and “What are you passionate about?” These edited video clips ranged in length from 14 to 127 s (*M* = 44 s). Target videos were compiled into eight subsets of 9–15 targets for viewing by perceivers. After viewing each video, perceivers rated targets on the abbreviated 21-item version of the BFI plus the three intelligence items.

##### Item observability

To determine whether IBC variability was related to expressive accuracy for more observable items specifically, a separate sample of 106 undergraduate students from the same population rated to what extent they agreed that each item was highly observable or visible on a 1 (*strongly agree*) to 7 (*strongly disagree*) scale. Examples of items that were rated higher in observability included items related to extroversion, such as (*Is full of energy*) and agreeableness (*Is helpful and unselfish with others*). Examples of items that were rated as lower in observability include items related to neuroticism (*Is depressed, blue*), other aspects of agreeableness (*Is forgiving*), and openness (*Is ingenious*).

#### IBC

**Figure 2.**
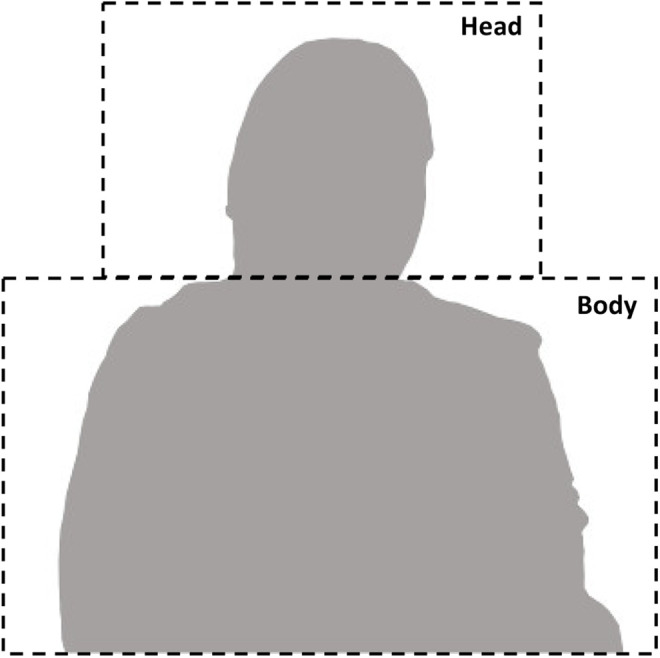
For each video, regions of interest were drawn around the head and the body regions of the participants. *Note*. Motion within those regions was calculated using optical flow analysis and then correlated using Correlation Map Analysis (Barbosa et al., 2012) to determine the amount of movement coordination between the region.

To determine IBC, the coordination between head and body movements was analyzed using Correlation Map Analysis (CMA; Barbosa et al., 2012; see SOM for complete technical details regarding this method). CMA is a two-step method that calculates the relationship between two motion signals. In the first step, optical flow analysis (OFA) or the pattern of movement of objects, edges, and surfaces is computed based on differences in pixel intensity from one to the next, where a greater change is indicative of greater motion. By summing the motion within a given region of interest (ROI) at each frame, a time series of total motion within that region is identified. Here, we drew static ROIs around participants’ head and body such that they encompassed the full range of motion of the two regions and calculated the magnitude of motion within those regions using OFA (Figure 2).^5^

In the second step of this analysis, the time series of total motion within each ROI were correlated using CMA. CMA utilizes a moving filter to calculate the instantaneous correlation between the motion in a given ROI and the motion in another ROI. In other words, motion differences within a small “window” of frames within one ROI can be compared to the same “window” of frames in the other ROI, by correlating the motion values frame-by-frame along the entire length of the video. In this manner, we can examine the coordination of motion between the head and body regions of the targets during their interview. A high correlation indicates greater coordination between the head and body region, or greater IBC, while a low correlation indicates lower coordination between the regions or lower IBC.^6^

This analysis yielded two measures of IBC. Here, the *average IBC level*, for each target, was calculated by finding the average correlation between the head and body ROIs across the length of the entire interview (*M* = 0.75, *SD* = 0.09).^7^ The *variability in*, for each target, was determined by calculating the standard deviation of the correlation between the two regions across the entirety of the interview (*M* = 0.19, *SD* = 0.06). As noted above, given the high correlation between these two values, *r* = −.68, *p* < .001, and additional analyses indicating IBC variability was more robustly associated with expressive accuracy (see SOM), we focus on IBC variability below.^8^

### Analytical Approach for Expressive Accuracy

To examine distinctive accuracy and how it was predicted by IBC levels and variability, we used the social accuracy modeling procedures (Biesanz, 2010) with the lme4 multilevel modeling package in R (R Development Core Team, 2009; Bates et al., 2015). The full equations are available in SOM, and the data and R code to recreate the analyses, as well as all of the output, are provided on Open Science Framework (https://osf.io/kwsjv/?view_only=26807d086fe743288aaa66baa8b9506e). Briefly, in Level 1 of the model, we predicted the perceivers’ rating of each target on each item from both the target’s accuracy criterion (averaged self and close-other ratings) on that item (to assess distinctive accuracy) and the mean self-report of all targets on that item (to assess normative accuracy). To enhance model convergence and interpretability (Biesanz, 2019), the mean participant self-report (normative criterion) was subtracted from the distinctive accuracy criterion prior to analyses. Slopes for both distinctive and normative accuracy were allowed to vary randomly for both targets and perceivers.^9^ The profile of observability ratings for each item was also included at Level 1 and included as a moderator of distinctive and normative accuracy slopes to confirm previous findings that distinctive accuracy tends to be higher for more observable items.

To examine whether IBC variability relates to target expressive accuracy, at Level 2 of the model, we included IBC variability as a predictor of the distinctive and normative accuracy slopes and their interactions with item observability. First, a significant three-way interaction between item observability, IBC variability, and the distinctive accuracy criterion predicting perceivers’ impressions would indicate that the association between IBC variability and distinctive accuracy differs as a function of item observability. If so, the interaction between target IBC variability and distinctive accuracy will be examined at high (1SD above the mean) and low (1SD below the mean) levels of item observability. For example, if for high observability items, there was a significant positive interaction between IBC variability and target distinctive accuracy, this would indicate that the perceivers’ impressions of the targets on high observability items were more in line with the target’s unique personality profiles when targets’ IBC variability was higher. Associations with normative accuracy were also examined and are reported in the SOM.

There is no established method for computing effect size estimates for the Level 1 effects. However, we provide effect size estimates and 95% confidence intervals for the key associations between IBC levels and variability and accuracy. Effect sizes were computed as the change in distinctive accuracy for a 2-standard deviation increase in the predictor of interest, which makes these estimates comparable to Cohen’s *d* (Gelman, 2008). Given the large number of dyadic-level observations, we used the “Wald” method in the *lme4* package to obtain 95% confidence intervals (see Human et al., 2020).

## Results

### Accuracy Levels and Item Observability

On average, perceivers were able to accurately judge targets’ unique profiles of personality items, viewing targets’ personalities with significant levels of distinctive accuracy, *b* = .12, *z* = 5.02, *p* < .001. In addition, in line with past work, there was a significant interaction with item observability, *b* = .46, *z* = 6.13, *p* < .001, such that distinctive accuracy was significantly greater for higher observability items (1 *SD* above the mean), *b* = .16, *z* = 6.48, *p* < .001, than low observability items (1 *SD* below the mean), *b* = .08, *z* = 3.25, *p* < .01, although accuracy was still statistically significant. Perceivers also viewed targets significantly in line with the normative profile at mean levels of item observability, *b* = .64, *z* = 14.91, *p* < .001, but less so on high observability items, *b* = .43, *z* = 9.43, *p* < .001, than low observability items, *b* = .85, *z* = 19.20, *p* < .001, interaction, *b* = −.25, *z* = −16.05, *p* < .001.

### IBC Variability and Accuracy

**Figure 3.**
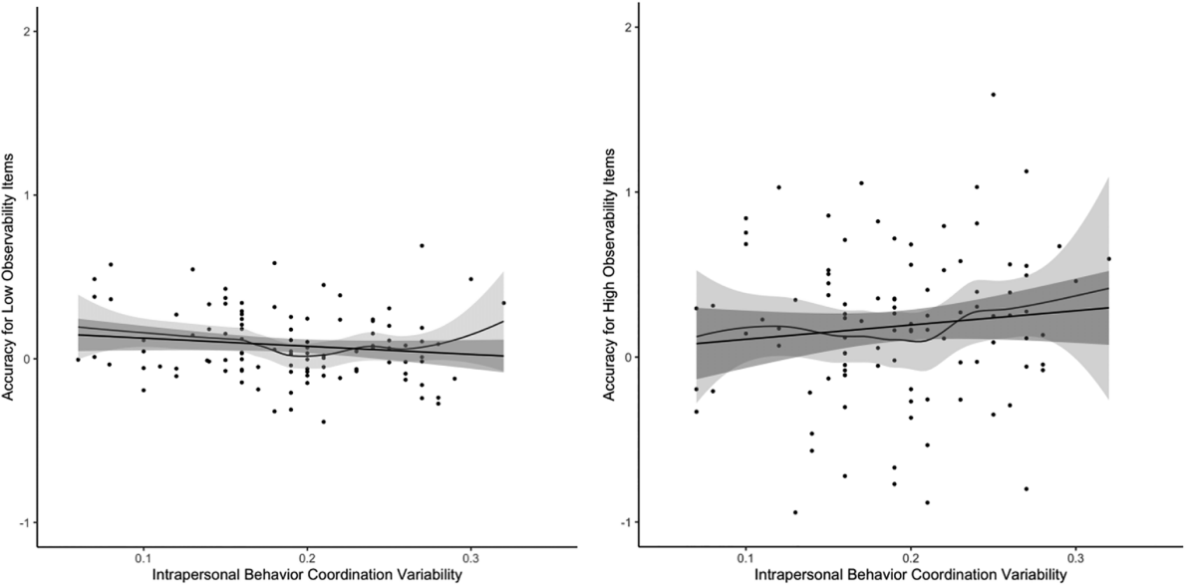
The relationship between intrapersonal behavioral coordination (IBC) variability and expressive accuracy for low and high observability personality items. *Note*. Expressive accuracy values are the empirical Bayes estimates for targets from models including either low or high observability items (items below or above the mean on observability), respectively. As such, the scores represent an approximation of the relationship between expressive accuracy and IBC variability at different levels of item visibility, rather than a direct representation. IBC variability values are each target’s standard deviation of the correlation between movement in the head and body regions across the entirety of a video-recorded interview. Both the linear slope (black line) and nonparametric loess relationship (dark gray curved line) with 95% confidence interval bands are plotted. Please note that the linear slope is an approximation of the results described in the text, because to obtain the linear slope, we used saved out expressive accuracy scores from models based on either only low observability items or high observability items (those below or above the mean observability ratings), which were then predicted by IBC variability scores in a linear regression model.

We next examined the role of variability in IBC, first examining whether the associations between target’s IBC variability and accuracy differed as a function of item observability. There was a significant three-way interaction with item observability, *b* = .05, *z* = 7.18, *p* < .001, such that greater IBC variability was associated with significantly greater distinctive accuracy for high observability items, *b* = .07, *d* = .61, 95% CI [0.20, 1.03], *z* = 2.91, *p* < .01, and was not significantly associated with distinctive accuracy for low observability items, *b* = −.02, *d* = −.19, 95% CI [−0.61, −0.23], *z* = −0.87, *p* = .39 (see Figure 3). IBC variability was not significantly associated with distinctive accuracy at mean observability levels or when the interaction with the observability profile was not included in the model, all *p*s > .30.

Specifically, for high observability items, targets who exhibited high IBC variability (1 *SD* above the mean) were viewed more accurately on more observable personality items (*b* = .23, *z* = 6.67, *p* < .001) than targets low in IBC variability (1 *SD* below the mean; *b* = .09, *z* = 2.63, *p* < .01), though these targets were still seen with significant levels of accuracy on these items.

As previously noted, IBC variability was also significantly positively correlated with several indicators of expressivity and social skill, some of which were also associated with expressive accuracy in a similar fashion (see SOM). Nevertheless, the significant interaction between target IBC variability and item observability predicting higher distinctive accuracy slopes held controlling for each of these correlates, as well as video length, whereas many of these correlates were no longer associated with expressive accuracy controlling for IBC variability (see SOM for full details).

## Discussion

In this study, we examined whether expressive accuracy—being viewed more in line with one’s unique personality profile—was related to a novel concrete aspect of nonverbal behavior that may indicate greater nonverbal expressivity and social communication skill: the variability in the coordination between a person’s head and body movements (IBC variability). Our data revealed that IBC variability was related to greater expressive accuracy specifically on more observable personality items, such as how energetic or helpful a person is. In contrast, IBC variability was not significantly associated with expressive accuracy on less observable items, such as how depressed or forgiving a person is. Importantly, this association held controlling for other indicators of motion, like greater variability in head movements alone, and relevant traits such as self- and perceiver-reported extroversion. Thus, IBC variability may play an important role in accurately expressing one’s personality to others, although these results need to be replicated to determine their robustness.

Why would IBC variability promote expressive accuracy? One possibility is that IBC variability is an indicator of making more relevant cues to observable personality items available to others. That is, when people coordinate their head and body in a more variable fashion, this may provide perceivers with more information about their personality. For example, when targets sometimes move their hands as much as their heads and other times less so, they may be varying between emphasizing important information (e.g., being both verbally and nonverbally expressive with both frequent mouth and hand movements), and providing additional, complementary information (e.g., saying less but gesturing more to compensate), ultimately conveying information more effectively than if body and head motion are more consistently coordinated, and thus perhaps more redundant. Indeed, IBC variability was negatively correlated with levels of IBC, and greater IBC levels were associated with lower expressive accuracy for high observability items when examined alone (see SOM). This suggests that lower IBC variability tended to be characterized by more consistently coordinated movements. IBC variability was also correlated with more movement overall, given the positive correlations with both levels and variability in head and body motion (see SOM). Interestingly, only IBC variability remained significant when both were examined together (see SOM). This suggests that it is not greater movement or variability per se, but variability in IBC specifically that may convey the most information to perceivers. This is also consistent with IBC variability being linked to expressive accuracy above and beyond self-reported and perceiver-rated characteristics that may also suggest greater expressivity, such as extroversion and engaging behavior, but that do not necessarily capture such a specific aspect of nonverbal communication. Thus, greater variability in IBC may be a unique nonverbal indicator of expressive accuracy in first impressions through its influence on cue relevance and availability.

Another possibility is that IBC variability modulates attentional processes, influencing the cue detection stage of RAM. In general, variability in movement patterns leads to greater attention by observers and within socio-communicative contexts, influencing how attention is allocated when two individuals interact (Mancas et al., 2008; Pashler et al., 2001). It is possible that when people vary how their head and body move in relation to one another, attention is directed to and engaged with this change more so than in cases of more consistently coordinated behavior, that is, low IBC variability. This is consistent with our findings that greater IBC variability is associated with being perceived as more engaging, genuine, and likable. Given that greater attentional engagement is linked to forming more accurate impressions (Capozzi et al., 2020), qualities of targets that draw perceivers in could enhance accuracy. That greater IBC variability only enhanced accuracy for high observability items is consistent with the idea that greater attention will only facilitate accuracy for traits that have clear behavioral manifestations (Human & Mendes, 2018) and the multiplicative nature of RAM (Funder, 1995). Examining how observers allocate their attention to targets high and low in IBC variability warrants further investigation.

Given that the present study was cross-sectional, it will be important for future work to examine the causal associations between IBC variability and accuracy and investigate these possible mechanistic pathways. A related outstanding question is whether targets can actively control their IBC variability to influence how accurately they are being perceived, which may be more difficult to modify, particularly for longer periods of time.

One factor influencing the interpretability of these findings concerns the role of verbal content of the targets’ speech on expressive accuracy, given that perceivers had access to both verbal and nonverbal information from targets. It is not clear if IBC variability would be similarly associated with expressive accuracy if verbal information was not provided. Perhaps IBC variability facilitates expressive accuracy because of the complementary role that such nonverbal behaviors could play with verbal content and may therefore be less relevant if only nonverbal behavior was provided. This is an interesting question for future research. However, given that most naturalistic social interactions do involve both verbal and nonverbal content, the current design provides insight into how these processes may be linked in a more ecologically valid setting.

In sum, the present study demonstrates that variability in IBC between head and body movements is related to the accuracy of personality expression. We found that greater variability in IBC was related to forming more accurate impressions of targets’ more observable characteristics, such as their tendencies to be energetic and helpful. As such, variability in IBC may represent an important behavioral marker of being a good target, potentially indicating how much and how well a person expresses themselves to others. The work presented here lends itself to future research to, first and foremost, confirm these findings by replicating them with a separate set of targets in a similar context, as well as to better understand the causal associations between IBC variability and expressive accuracy, and to examine the generalizability of these links between IBC and accuracy in more diverse samples and different social contexts.

## Supplemental Material

Supplemental Material, sj-docx-1-spp-10.1177_19485506211011317 - Intrapersonal Behavioral Coordination and Expressive Accuracy During First Impressions Click here for additional data file. Supplemental Material, sj-docx-1-spp-10.1177_19485506211011317 for Intrapersonal Behavioral Coordination and Expressive Accuracy During First Impressions by Nida Latif, Lauren J. Human, Francesca Capozzi and Jelena Ristic in Social Psychological and Personality Science

## Notes

1. Specifically, Capozzi et al. (2020) examined how perceiver attentional engagement, assessed with eye tracking, was related to distinctive and normative accuracy, using a subsample of eight targets and a different set of perceivers. The research question and analyses do not overlap.

2. This study involved an experimental manipulation aimed at increasing how accurately targets expressed themselves, but this did not influence the results presented here. A manuscript examining the effects of this manipulation on target expressive accuracy, perceiver liking, and target state well-being is currently in preparation (Mignault et al., in prep). The research questions and analyses do not overlap. Targets and perceivers also completed other measures that are not related to the current research questions. A full list of measures can be found on the Open Science Framework (https://osf.io/kwsjv/?view_only=26807d086fe743288aaa66baa8b9506e).

3. A total of 108 targets completed the study, of which one excluded because they did not consent to having their video shown to future participants and two because their videos failed to record.

4. Note that this power analysis was not based on testing the key three-way interaction between intrapersonal behavioral coordination (IBC) variability, item observability, and expressive accuracy and is therefore an approximation that could have overestimated power.

5. Since the analysis relies on *change* in pixels, the pixels comprising the background do not factor in this calculation of motion. Magnitude of motion is expressed with the unit “pixels/frame,” indicating the change in pixels from one frame to the next.

6. In this analysis, a positive correlation would represent both regions moving similarly in the same horizontal and vertical direction, while a negative correlation would represent two regions moving in the opposite horizontal and vertical directions. For example, a negative correlation might emerge if the head moved up and to the right while the arm simultaneously moved down and to the left.

7. No transformations were applied to average correlations since the time series are weighted by a Gaussian function.

8. The stability of these measures was examined by correlating these values for the first half and the second half of all the videos. This revealed a correlation of *r*(103) = .83, *p* < .0001, for levels and a correlation of *r*(103) = .79, *p* < .0001, for variability suggesting that our measures had good stability.

9. In line with prior work, for parsimony and to reduce model convergence issues, we did not include dyadic random effects in the primary models (e.g., Rogers & Biesanz, 2019), but all results held with dyadic effects included.
