# Supplementary material for: Intrapersonal Behavioral Coordination and Expressive Accuracy During First Impressions
Source: Soc Psychol Personal Sci. 2021 Apr 28;13(1):150–9. doi: 10.1177/19485506211011317 (PMC8652366; doi:10.1177/19485506211011317)
Supplement: Supplemental Material, sj-docx-1-spp-10.1177_19485506211011317 - Intrapersonal Behavioral Coordination and Expressive Accuracy During First Impressions [file sj-docx-1-spp-10.1177_19485506211011317.docx]

**Supplementary Online Materials**

**Analytical Approach: Model Equations**

Elaborating on the description provided in the manuscript, here we provide the equations and more detailed explanation of the coefficients included within social accuracy model (SAM; Biesanz, 2010, 2020). The standard level-1 SAM equation for assessing distinctive and normative accuracy is as follows:

$Y_{pti}=\beta_{0pt}+\beta_{1pt}{TargetPersonality}_{ti}$+ $\beta_{2pt}{NormativeMean}_{i}+ \varepsilon_{pti}$ (1.1)

$\beta_{0pt}=\beta_{00}+u_{0p}+ u_{0t}$

$\beta_{1pt}=\beta_{10}+u_{1p}+ u_{1t}$ (1.2)

$\beta_{2pt}=\beta_{20}+u_{2p}+ u_{2t}$

In this model, $Y_{pti}$is Perceiver *p*’s rating of Target *t* on item *i*. ${TargetPersonality}_{ti}$is Target *t*’s distinctive accuracy criterion (the target’s self-report or composite self and informant(s) report) on item *i* after subtracting the normative profile (${NormativeMean}_{i})$. Thus, $\beta_{1pt}$is the regression coefficient for the distinctive accuracy slope: the association between Target *t*’s distinctive accuracy criterion on item *i* predicting Perceiver *p*’s rating of Target *t* on the same item *i*. ${NormativeMean}_{i}$ is the average target self-report on each item *i*. As such, $\beta_{2pt}$is the regression coefficient for the normative accuracy slope: the relationship between the mean target self-report for item *i* predicting Perceiver *p*’s rating of the same item *i*. The average levels of distinctive accuracy and normative accuracy are reflected by _
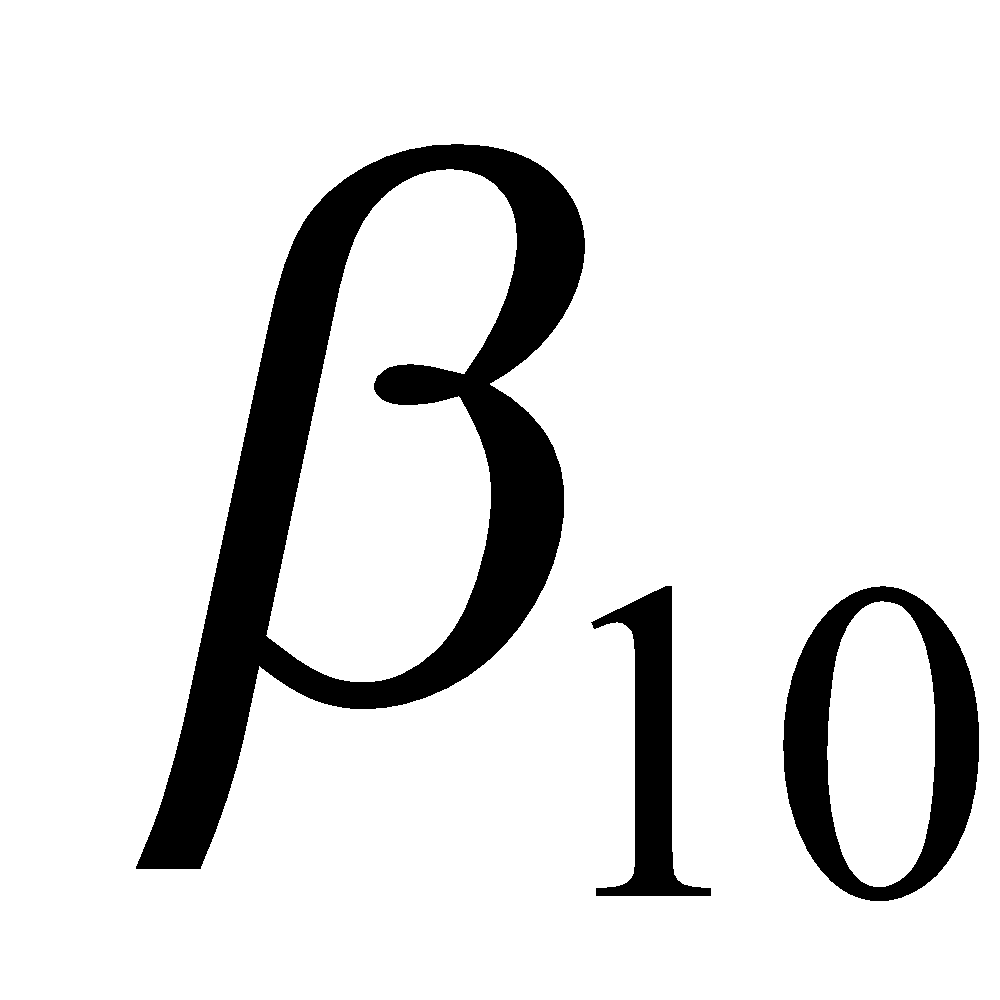
_ and _
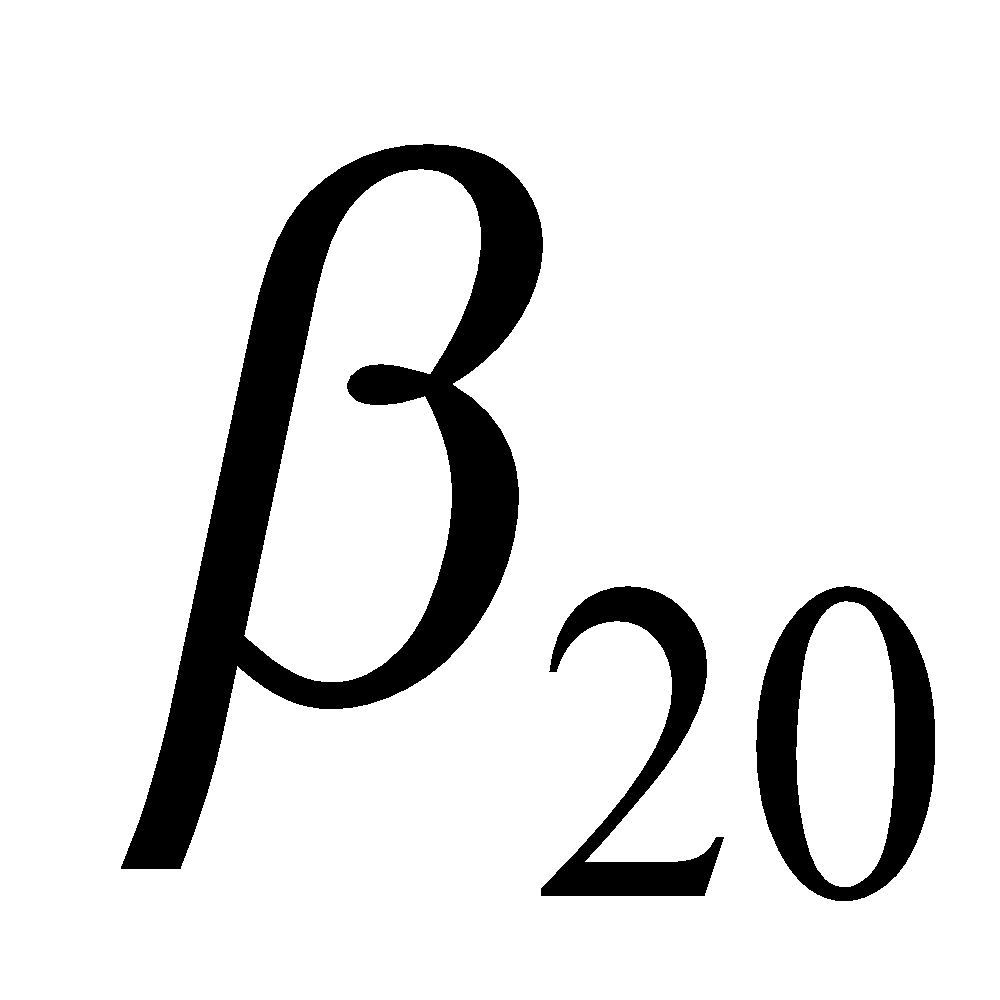
_, respectively. $\beta_{0pt}$ is the intercept, the expected value of perceiver *p*’s rating of target *t* on item *i* when the accuracy criterion and normative personality criterion are held constant at their means. It does not have a conceptual meaning given the structure of the data. In Equation 1.2, $u_{1p}$ and $u_{1t}$represent the variation due to perceivers and targets around distinctive accuracy levels (i.e., distinctive accuracy random affects) and$u_{2p}$ and $u_{2t},$represent the variation due to perceivers and targets around normative accuracy levels (i.e., normative accuracy random affects). These are not discussed in the present manuscript but were very similar in magnitude to previous research (e.g., Human et al., 2021).

To examine the role of IBC variability in expressive accuracy, target IBC variability was included as a predictor of both the distinctive and normative accuracy slopes at Level 2 of the model, as shown with the following equation:

$\beta_{0pt}=\beta_{00}+ \beta_{01}{IBCVariability}_{pt}+u_{0p}+ u_{0t}$

$\beta_{1pt}=\beta_{10}+ \beta_{11}{IBCVariability}_{pt}+u_{1p}+ u_{1t}$ (2)

$\beta_{2pt}=\beta_{20}+ \beta_{21}{IBCVariability}_{pt}+u_{2p}+ u_{2t}$

Here, $\beta_{11}$ is the interaction between Target *t*’s IBC variability and the Target *t*’s personality criterion predicting Perceiver *p*’s ratings. This interaction demonstrates whether IBC variability was higher when the target’s distinctive personality profile was viewed more in line with perceiver ratings (i.e., distinctive accuracy) – that is, do perceivers view a target more in line with their self- and close-other reports when the target exhibits more variable coordination between their head and body movements? $\beta_{21}$ is the interaction between IBC variability and the mean target self-report predicting perceiver ratings, which demonstrates whether IBC variability was higher when the normative personality profile is more line with perceiver ratings (i.e., normative accuracy), although this is not of primary interest in the present manuscript.

In the present study, we expanded the SAM to also include the mean item observability ratings at Level 1 as a predictor of perceiver ratings ($Y_{pti}$), the Level-1 distinctive and normative accuracy slopes ($\beta_{1pt}$ and $\beta_{2pt}$, respectively), and the Level-2 interactions between IBC variability and distinctive and normative accuracy ($\beta_{11}$ and $\beta_{21}$, respectively). This latter three-way interaction with distinctive accuracy tells us whether IBC variability relates to distinctive accuracy differently for items that are rated higher vs. lower in observability. If so, the simple effects between IBC variability and distinctive at high and low levels of item observability are presented as the key results.

**Technical Details for Correlation Map Analysis**

To determine intrapersonal behavioral coordination, the coordination between head and body movements was analyzed using Correlation Map Analysis (CMA; Barbosa et al., 2012). CMA is a two-step method that calculates the relationship between two motion signals. In the first step, optical flow, or the pattern of movement of objects, edges, and surfaces is computed based on the relative motion between an observer and the camera to quantify overall motion. This optical flow analysis (OFA) uses the Horn & Schunck method (1980), a computer vision technique in which patterns of pixel intensity are compared between consecutive video frames. The amount of motion change associated with each pixel from one frame to the next is calculated from intensities of the neighboring pixels. The differences in pixel intensities result in a series of pixel velocities corresponding to the magnitude of motion expressed in pixels/frame indicating the change in pixels from one frame to the next^[[1]](#footnote-1)^. By summing the motion within a given region of interest (ROI) at each frame, a time-series of total motion within that region is identified. Here, we drew static ROIs around participants’ head and body such that they encompassed the full range of motion of the two regions and calculated the magnitude of motion within those regions using OFA (Figure 2)^[[2]](#footnote-2)^.

In the second step of this analysis, the time-series of total motion within each ROI were correlated using CMA. CMA utilizes a moving filter to calculate the instantaneous correlation between the motion in a given ROI with the motion in another ROI. In other words, motion differences within a small ‘window’ of frames within one ROI can be compared to the same ‘window’ of frames in the other ROI along the entire length of the video. Here, we examined motion differences in a window that was 250ms in length^[[3]](#footnote-3)^. This means that that the motion values for all frames within that window for the head were correlated with the motion of all frames within that same window for the body. The whole window was moved over one frame and the process repeated until the motion of the entire length of the video was calculated, resulting in a time-series of correlation values. In this manner, we can examine the coordination of motion between the head and body regions of the targets during their interview. A high correlation indicates greater coordination between the head and body region, or greater IBC, while a low correlation indicates lower coordination between the region, or lower IBC^[[4]](#footnote-4)^.

**Correlates of IBC Variability**

We also examined the relationships between IBC variability and IBC levels and a number of other potential correlates, including overall motion and variability, target self-reported and perceiver-rated Big Five personality traits, and perceiver-reported social behaviors, including how engaging and likeable targets were, as well as video duration. We examined whether each was associated with IBC variability, as well as with expressive accuracy on their own and as a function of item observability. Finally, we included each as additional predictors of distinctive accuracy in the models with IBC variability as controls.

***IBC Level.*** As noted in the manuscript, IBC levels and variability were significantly correlated, *r* = -.68, *p* < .001. Examining IBC levels separately with expressive accuracy, we also found a significant interaction with item observability predicting distinctive accuracy slopes, although negative in this case, *b* = -.05, *z* = -6.15, *p* < .001. Simple slopes analyses revealed that greater IBC levels were associated with being seen significantly less accurately on high observability items, *b* = -.07, *z* = -2.86, *p* < .01, but not on low observability items, *b* = .01, *z* = 0.35, *p* = .73. Thus, targets who were generally less coordinated in their movements were seen with greater accuracy, suggesting that lower coordination may provide more information to perceivers, perhaps by providing more complementary as opposed to redundant information, and/or because less coordinated movements capture greater perceiver attention. However, as noted in the primary manuscript, when examined alongside IBC variability, this significant interaction between IBC levels, item observability, and distinctive accuracy was no longer significantly associated with expressive accuracy, whereas the interaction with IBC variability was not, suggesting that this link could be driven by the overlap with IBC variability.

***Overall motion***. Overall head and body motion levels and variability (calculated, respectively, as the average motion and the standard deviation of motion within each region over the entire length of the interview) were all significantly positively correlated with IBC variability, all *r*s > .28, all *p*s < .01 (see Table S1). None of these indicators were significantly related to accuracy on average across items but variability in head motion and level of body motion had significant interactions with trait observability predicting distinctive accuracy (all *p*s < .05). Examining the simple slopes, overall levels of body motion were positively related to accuracy for high observability items and negatively related for low observability items, but neither association was significant, all *p*s > .32. For head motion variability, like IBC variability, greater variability was significantly associated with greater accuracy for high observability items, *b* = .01, *z* = 2.42, *p* = .02, and not significantly related to accuracy for low observability items, *b* = -.004, *z* = -0.79, *p* = .43.

Importantly, the item observability and expressive accuracy interaction with IBC variability held controlling for both body motion levels and head motion variability, all *p*s < .001, and the simple association between greater accuracy for high observability items held for IBC variability, *b* = .01, *z* = 2.21, *p* = .03, but not head motion variability, *b* = .01, *z* = 1.48, *p* = .14, with both in the model. Overall, then, greater variability in expressive movement more generally is linked to greater expressive accuracy, but variability in head and body movement coordination appears to be a more robust correlate than general head motion variability.

***Self-reported Big Five Traits***. Targets’ standing on the Big Five traits were computed using scores from the full 44-item BFI. IBC variability was significantly correlated with self-reported extraversion, *r* = .21, *p* = .03, but no other personality traits, all *p*s > .41 (see Table S1). Target self-reported extraversion did not significantly predict expressive accuracy on average across items, *b* = -.01, *z* = -.42, *p* = .68, but, like IBC variability, did significantly interact with item observability to predict expressive accuracy, *b* = .02, *z* = 2.36, *p* = .02. Nevertheless, the three-way interaction between IBC variability, item observability, and expressive accuracy held controlling for self-reported extraversion (*b* = .05, *z* = 6.86, *p* < .001), whereas the interaction with self-reported extraversion did not (*b* = .00, *z* = 0.27, *p* = .79).

***Perceiver-rated Big Five Traits***. Perceiver-rated target standing on the Big Five traits were computed using available items for each trait on the 21-item version of BFI. Greater IBC variability was significantly associated with greater perceiver-rated extraversion and lower perceiver-rated neuroticism, all |*r*|s > .26, all *p*s < .01, but none of the other traits (see Table S1). Perceiver-rated extraversion significantly predicted greater expressive accuracy on average across items, *b* = .05, *z* = 2.09, *p* = .04, and significantly interacted with item observability to predict expressive accuracy, *b* = .01, *z* = 8.47, *p* < .001. The three-way interactions with item observability and expressive accuracy held for both perceiver-rated extraversion and IBC variability when both were included in the models, all *p*s < .001.

Perceiver-rated neuroticism did not significantly predict greater expressive accuracy on average across items, nor significantly interact with item observability to predict expressive accuracy, all *p*s > .42, and the three-way interaction with item observability, expressive accuracy, and IBC variability held controlling for perceiver-rated neuroticism.

***Perceiver-rated social behaviors.*** Perceivers also rated targets on several dimensions that could be considered relevant social behaviors, including how engaging, confident, genuine, and likeable they were, all rated on a 1 (*strongly agree*) to 7 (*strongly disagree*) scale. IBC variability was associated with being perceived as significantly more engaging, genuine, and likeable, and marginally more confident, all *r*s > .17, all *p*s < .08. Examining each with expressive accuracy, perceiver-reported target engagement, confidence, genuineness, and likeability were not significantly associated with greater expressive accuracy on average across items, all *p*s > .21, but each did significantly or marginally interact with item observability to predict expressive accuracy, all *p*s < .10. Even so, the three-way interactions with item observability, expressive accuracy, and IBC variability held controlling for each perceived behavior, all *p*s < .001, whereas most perceived behaviors no longer significantly interacted with item observability and expressive accuracy, with the exception of confidence, *b* = .02, *z* = 2.56, *p* = .01.

***Video duration***. Duration of video was significantly correlated with IBC variability, *r* = .21, *p* = .03, and significantly predicted greater distinctive accuracy on average across items and as a function of item observabilility, all *p*s < .01. Both video duration and IBC variability continued to significantly interact with item observability and expressive accuracy when in the model together (all *p*s < .001).

**Summary**. Taken together, the pattern of correlations with IBC variability provide support for the idea that greater IBC variability reflects greater nonverbal expressivity and social communication skill. Specifically, people higher in IBC variability tend to move their heads and bodies more frequently and variably in general, report being more extraverted, and are perceived to be more extraverted, engaging, genuine, and likeable. However, although some of these correlates of IBC variability showed similar associations with expressive accuracy, the unique association between IBC variability and expressive accuracy for high observability personality items was robust controlling for each one. As such, these correlates help us to shed conceptual light on what it means to display higher IBC variability – specifically, a more nonverbally expressive, socially skillful, engaging nonverbal communication style – yet do not fully account for why IBC variability is related to greater expressive accuracy.

**Normative Accuracy**

Although this was not of primary interest, for completeness we also report the associations with normative accuracy. There was also a significant three-way interaction between item observability, IBC variability, and normative accuracy, *b* = .04, *z* = 2.37, *p* = .02, such that IBC variability was significantly associated with greater normative accuracy for high observability traits (*b* = .08, *z* = 2.73, *p* = .01) but not low observability traits (*b* = .02, *z* = 0.76, *p* = .45). Given the link between normative accuracy and social desirability, this is consistent with the finding that IBC variability was associated with greater perceived likeability. In other words, people who display greater IBC variability may be better liked and, correspondingly, their personalities are seen in a more normative, socially desirable manner.

| Potential Correlate | Descriptive Statistics | | Correlation with IBC Variability | |
| --- | --- | --- | --- | --- |
|  | *M* | *(SD)* | *r* | *[.95CI]* |
| Movement Indicator |  |  |  |  |
| Head Motion Level | 13.51 | (3.25) | .26** | [.07, .43] |
| Body Motion Level | 17.11 | (6.38) | .41*** | [.23, .55] |
| Head Motion Variability | 4.69 | (5.02) | .38*** | [.20, .53] |
| Body Motion Variability | 11.28 | (10.44) | .41*** | [.24, .56] |
| Self-Reported Traits |  |  |  |  |
| Extraversion | 4.40 |  | .21* | [.02, .39] |
| Neuroticism | 3.96 | (1.08) | -.01 | [-.20, .18] |
| Agreeableness | 5.24 | (0.86) | -.08 | [-.27, .11] |
| Conscientiousness | 5.00 | (0.87) | .02 | [-.18, .21] |
| Openness | 4.91 | (0.81) | -.05 | [-.24, .14] |
| Perceiver-rated Traits |  |  |  |  |
| Extraversion | 4.14 | (0.96) | .29** | [.10, .46] |
| Neuroticism | 3.40 | (0.36) | -.26** | [-.43, -.08] |
| Agreeableness | 4.90 | (0.45) | .16 | [-.03, .34] |
| Conscientiousness | 4.81 | (0.46) | .11 | [-.08, .30] |
| Openness | 4.40 | (0.56) | .09 | [-.10, .28] |
| Perceiver-rated social behavior |  |  |  |  |
| Engaging | 4.52 | (0.83) | .24* | [.05, .41] |
| Confident | 4.62 | (0.92) | .18† | [-.02, .36] |
| Genuine | 5.26 | (0.57) | .39** | [.22, .54] |
| Likeable | 5.02 | (0.67) | .30** | [.11, .46] |
| Video Duration | 43.82 | (16.79) | .21* | [.02, .39] |

Table S1. Descriptive statistics and associations with intrapersonal behavioral coordination variability for potential correlates.

*Note*. IBC = Intrapersonal behavioral coordination; M = mean; SD = standard deviation; r = Pearson correlation coefficient; .95CI = 95% correlation interval.

†*p* < .10, **p* < .05, ***p* < .01, ***p* < .001.

**Examples of High and Low Intrapersonal Behavioral Coordination (IBC) Variability**

| **** |
| --- |
| ***Figure 1.*** Examples of high and low IBC variability (Videos available at <https://osf.io/kwsjv/?view_only=26807d086fe743288aaa66baa8b9506e>). (A) A screenshot from a 10s video showing high intrapersonal coordination variability. Here, the coordination between the head and body varies frequently over the course of the video. The middle panel shows the movement of the head (solid grey line) and body (dashed black line). The bottom panel illustrate the coordination between the head and body regions. (B) A screenshot from a 10s video showing low intrapersonal coordination variability. Here, the coordination between the head and body does not vary frequently and remains high for the majority of the clip. *Note:* These video examples are included for illustrative purposes only using open-source videos in order to demonstrate extremes in IBC variability. The videos of actual targets used in the study could not be included due to privacy. Please note that while the total amount of motion may not be matched across the two example videos, the analyses in the manuscript controlled for the amount of motion in the video clips which were used in the study. |

1. The number of frames in a video is dependent on the frame rate of that video. For example, some videos have a frame rate of 30 frames/second. If there is a change of 10 pixels/frame, this would be 300 pixels/second and would indicated that in one second, 300 pixels of the video have changed. [↑](#footnote-ref-1)
2. Since the analysis relies on *change* in pixels, the pixels comprising the background do not factor in this calculation of motion. [↑](#footnote-ref-2)
3. This window size was based on values used by Danner et al., 2018 to reflect that fine-grained analysis appropriate for head-gesture coupling. The coupling between head and body movements is very tight, with body movements accompanying head movements at a timescale of 200ms (Pouw et al., 2020). Thus, our window size is sufficient to capture this coupling. [↑](#footnote-ref-3)
4. In this analysis, a positive correlation would represent both regions moving similarly in the same horizontal and vertical direction while a negative correlation would represent two regions moving in opposite horizontal and vertical direction. For example, a negative correlation might emerge if the head moved up and to the right while the arm simultaneously moved down and to the left. [↑](#footnote-ref-4)
